# Comparison of Effectiveness Between Ultrasound-Guided and Blind Corticosteroid Injections in Plantar Fasciitis: A Systematic Review and Meta-Analysis

**DOI:** 10.3390/life15071107

**Published:** 2025-07-15

**Authors:** Hoa Ngan Doan, Yoo Jin Choo, Min Cheol Chang

**Affiliations:** Department of Physical Medicine and Rehabilitation, College of Medicine, Yeungnam University, Daegu 42415, Republic of Korea; hoadn.hmu@gmail.com (H.N.D.); cyj361@hanmail.net (Y.J.C.)

**Keywords:** plantar fasciitis, ultrasound-guided injection, blind injection, corticosteroid injection, tenderness threshold, plantar fascia thickness

## Abstract

The effectiveness of ultrasound (US)-guided compared with blind corticosteroid injections for the treatment of plantar fasciitis (PF) remains uncertain. This meta-analysis aimed to evaluate the clinical benefits of US-guided over blind injections in patients with PF. A systematic search of PubMed, Embase, Web of Science, and Scopus was conducted, collecting articles published up to 20 April 2025. Randomized controlled trials comparing US-guided and blind corticosteroid injections for PF were included. The extracted outcome measures, i.e., visual analog scale (VAS), heel tenderness index (HTI), tenderness threshold (TT), and plantar fascia thickness, were assessed at short- (2–6 weeks) and long-term (≥12 weeks) follow-ups. Compared with the blind injection group, the US-guided group showed significantly greater improvement in TT at both short- and long-term follow-ups, as well as a greater reduction in plantar fascia thickness. However, no significant differences were found between the two groups in VAS and HTI scores. US-guided corticosteroid injections provide superior clinical benefits compared with blind injections in patients with PF, particularly in enhancing mechanical pain tolerance and reducing plantar fascia thickness. Nevertheless, these findings should be interpreted with caution due to the limited methodological quality of the included studies.

## 1. Introduction

Plantar fasciitis (PF), a leading cause of heel pain in adults [1], accounts for approximately 11–15% of foot complaints requiring professional care [2]. Despite often being idiopathic, the underlying pathophysiology of PF typically involves repetitive microtrauma at the enthesis of the plantar fascia on the calcaneus, causing degenerative rather than inflammatory changes [3,4]. Consequently, some experts refer to the condition as “plantar fasciosis” to emphasize its chronic degenerative nature rather than an acute inflammation. Patients with PF usually exhibit sharp, stabbing heel pain, which is most intense during the first steps after waking or following extended periods of rest [5,6]. This pain can impair daily activities such as standing and walking. Additionally, it may lead to compensatory gait changes that contribute to secondary musculoskeletal pain in the knees, hips, or lower back.

Initial treatment options for PF include activity modification, analgesics, rehabilitation exercises, and in-shoe orthotics [3]. For patients who do not respond to these measures, corticosteroids can be injected at the calcaneal insertion of the plantar fascia to relieve pain [7]. Corticosteroid injections are widely used to manage PF, effectively reducing pain scores within 4–12 weeks following an injection [8]. Achieving accurate delivery of corticosteroids into the pathological area of the plantar fascia is critical, as improper injection can increase the risk of complications such as spontaneous fascial rupture [9,10]. In clinical practice, corticosteroid injections for PF are generally administered using blind (palpation-guided), ultrasound (US)-guided, or scintigraphy-guided approaches. Scintigraphy can theoretically aid in localizing the areas of increased metabolic activity within the PF [11] to achieve precise injections. However, it is not widely available in most clinical settings and involves patient radiation exposure, limiting its routine application. Thus, blind and US-guided injections are more commonly utilized.

Blind injections depend on anatomical knowledge and palpation to identify landmarks but are inherently subject to both inter- and intra-operator variability, which can result in inconsistent needle placement and therapeutic outcomes. By contrast, US provides real-time visualization of the plantar fascia and surrounding structures, which may improve needle placement and therapeutic efficacy [12]. This concept is supported by Kane et al. [13], who showed four patients (five heels) that had failed to respond to blind corticosteroid injections but experienced successful outcomes following US-guided injections. Kane et al. [13] also concluded that US-guided injection provides a favorable clinical response in cases of failed blind injections. Tsai et al. hypothesized that the increased precision of US-guided injections achieves better clinical outcomes compared with blind injections [14].

Although several studies compared US-guided and blind corticosteroid injections for PF management, the findings obtained were inconsistent. Chen et al. conducted a randomized controlled trial (RCT) demonstrating that US-guided corticosteroid injections resulted in better therapeutic outcomes than blind corticosteroid injections [15]. In contrast, Saba et al. [16] and Kane et al. [17] did not find a clear advantage of US-guided injections over blind injections. Therefore, we conducted a meta-analysis to quantitatively assess the comparative effectiveness of US-guided versus blind corticosteroid injections in patients with PF.

## 2. Materials and Methods

### 2.1. Search Strategy

This systematic review was conducted according to the Cochrane Handbook for Systematic Reviews of Interventions. A comprehensive search was performed in PubMed, Embase, Web of Science, and Scopus for relevant studies published up to 20 April 2025. The search strategy comprised the following key terms: [(plantar fasciitis OR plantar fascia OR plantar fasciopathy OR heel OR heel pain) AND (ultrasound OR ultrasonography OR sonography OR ultrasound-guided)] AND [(ultrasound-guided injection OR injection OR corticosteroid injection OR plantar fascia injection OR corticosteroid) OR (saline solution OR placebo OR local anesthetics OR orthobiologics OR platelet-rich plasma OR whole blood OR mesenchymal stem cells OR amnion OR adipose tissue OR fat injection OR dextrose OR botulinum toxins OR fasciotomy OR tenotomy OR Tenex OR Prolotherapy OR electrolysis OR high-energy shock waves OR palpation OR anatomic landmarks OR physical therapy)] (Supplementary Materials S1). Filters were applied to limit results to studies only involving human participants.

### 2.2. Selection Criteria

Studies were included if they (1) enrolled adults diagnosed with PF; (2) compared US-guided corticosteroid injection with palpation-guided or blind corticosteroid injection; and (3) were published in English.

The exclusion criteria were (1) review articles, case reports, study protocols, conference abstracts, or other studies of non-original research formats; (2) animal studies; and (3) studies lacking sufficient data for analysis.

### 2.3. Data Extraction

All search results were imported into EndNote X9 for reference management. Duplicate entries were removed using the built-in deduplication function of the software. Two reviewers (DNH and MCC) independently screened the remaining articles by title and abstract based on the predefined inclusion criteria. The full-text versions of potentially eligible studies were assessed to confirm inclusion. Any discrepancies were resolved through discussion and consensus by involving a third reviewer (YJC) when necessary.

Data were extracted from studies of patients who received corticosteroid injections near the origin of the plantar fascia either under US guidance (US-guided injection group) or using a palpation-based approach (blind injection group). For variables reported in more than one study, meta-analyses were performed to compare outcomes between groups. Post-treatment follow-up was reviewed to identify overlaps, and outcomes were categorized as short-term (2–6 weeks after treatment) and long-term (≥12 weeks after treatment). When multiple measurements were available within a particular timeframe, the most recent value was used for analysis to better reflect a sustained intervention effect.

Extracted data comprised the first author, publication year, study design, number of treated feet, follow-up duration, and outcome measures. Treatment outcomes were evaluated using the visual analog scale (VAS), heel tenderness index (HTI), tenderness threshold (TT), and plantar fascia thickness. All outcome data were extracted as means and standard deviations.

The VAS is a patient-reported measure of pain intensity, typically ranging from 0 (no pain) to 10 (worst imaginable pain) [18,19]. In studies that reported VAS scores on a 100-point scale, values were converted to a 10-point scale for consistency. The HTI assesses heel pain upon palpation of the medial calcaneal tuberosity, being scored as 0 = no pain, 1 = painful, 2 = painful with wincing, and 3 = painful with wincing and withdrawal [17,20]. The TT represents the minimum pressure that induces pain when applied to the medial calcaneal tuberosity using a pressure algometer [14,15,21]. Plantar fascia thickness was measured at its proximal insertion at the medial calcaneal tubercle on a longitudinal view [22].

### 2.4. Methodological Quality Assessment

The methodological quality of the included studies was evaluated using the risk of bias domains outlined in the Cochrane Handbook for Systematic Reviews of Interventions, aiming to identify potential sources of bias. The following domains were assessed: (1) random sequence generation and allocation concealment (selection bias); (2) blinding of participants and personnel (performance bias); (3) blinding of outcome assessment (detection bias); (4) incomplete outcome data (attrition bias); (5) selective reporting (reporting bias); and (6) other potential sources of bias. Two independent reviewers (DNH and MCC) conducted the assessments. Discrepancies were resolved through discussion and consensus by involving a third reviewer (YJC) when necessary.

### 2.5. Statistical Analysis

Meta-analyses were performed using Review Manager (RevMan) version 5.3 (The Cochrane Collaboration, Copenhagen, Denmark). Heterogeneity among studies was assessed using the I^2^ statistic, which quantifies the degree of inconsistency across study results. An I^2^ value ≤ 50% indicated low heterogeneity, and a fixed-effect model was utilized. When I^2^ exceeded 50%, indicating substantial heterogeneity, a random-effect model was used. Because the data were continuous variables, results were reported as standardized mean differences (SMDs) with corresponding 95% confidence intervals (CIs). *p*-values < 0.05 indicated statistical significance. Forest plots were generated for all outcomes.

Both funnel plots and Egger’s tests were used to assess publication bias. These analyses were performed using R software (version 4.1.2, R Foundation for Statistical Computing, Vienna, Austria). Funnel plots were used to visually assess potential publication bias based on pooled effect estimates. Egger’s test was performed to statistically evaluate funnel plot asymmetry only when at least three studies were included in the meta-analysis. *p*-values < 0.05 according to Egger’s test were considered evidence of potential publication bias.

## 3. Results

### 3.1. Study Selection

**Figure 1:**
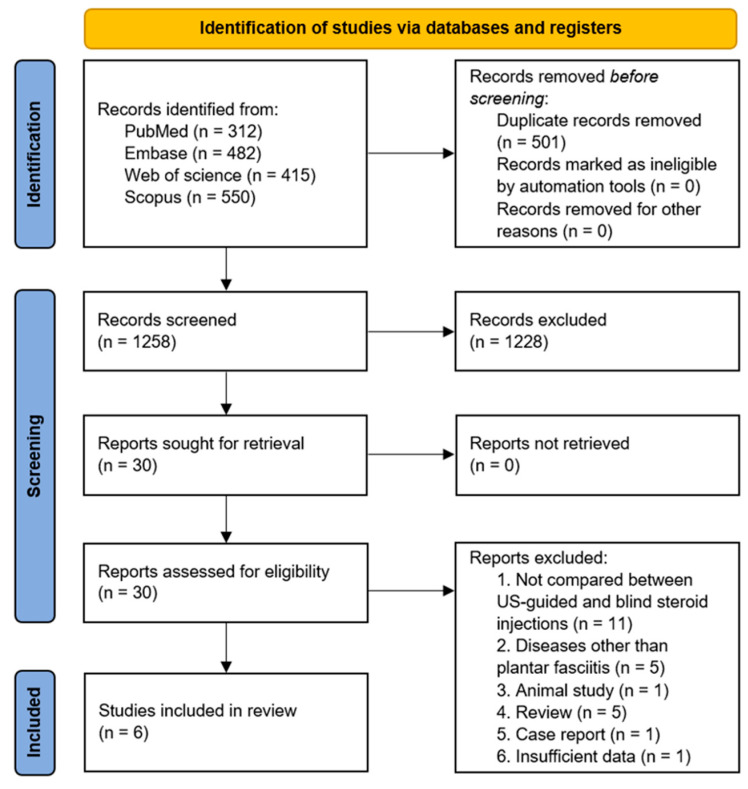
Flowchart of the study selection process for the meta-analysis.

In total, 1759 articles were identified, and 501 duplicates were removed (Figure 1). After screening titles and abstracts, 30 articles were selected for full-text reviews. Following a detailed evaluation, 24 articles were excluded for the following reasons: not a direct comparison between US-guided and blind corticosteroid injections (*n* = 11), unrelated diagnoses (*n* = 5), animal study (*n* = 1), review article (*n* = 5), case report (*n* = 1), and insufficient data (*n* = 1). Ultimately, six studies (RCTs) met the inclusion criteria and were included in the meta-analysis (Table 1) [14,15,16,17,20,23].

### 3.2. Study Characteristics

The six included studies [14,15,16,17,20,23] involved 89 treated feet in the US-guided injection group and 83 treated feet in the blind injection group. Table 1 summarizes detailed characteristics of the included studies.

### 3.3. Assessment of Study Quality

**Figure 2:**
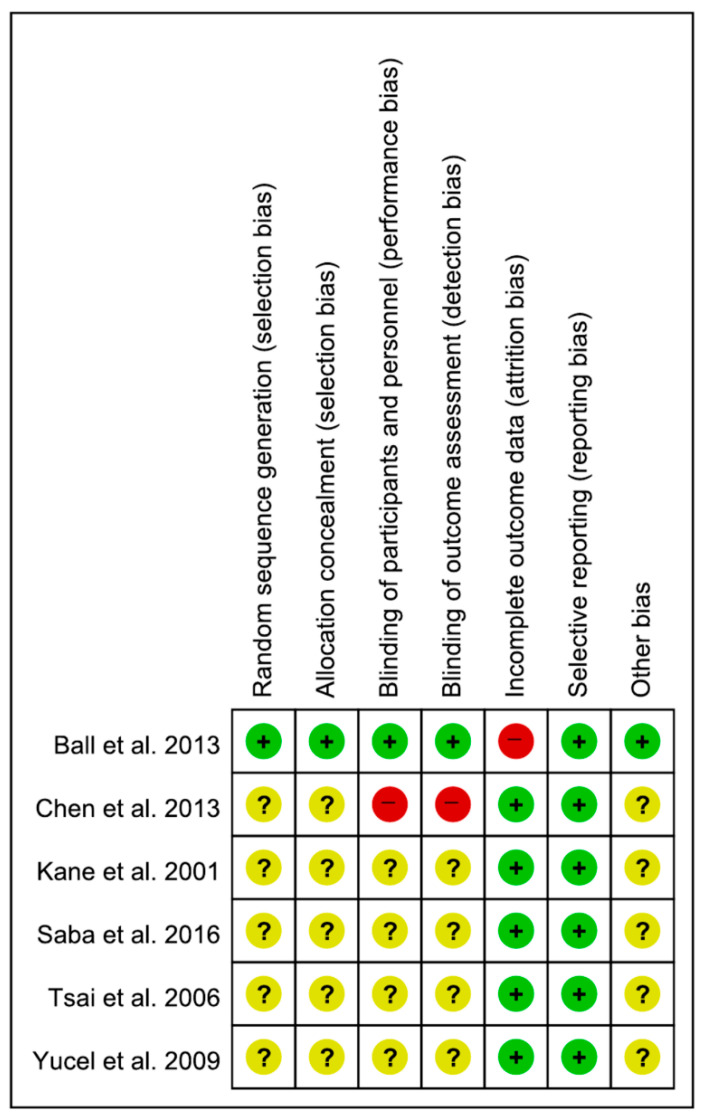
Summary of risk of bias assessment. +: low risk of bias, −: high risk of bias, ?: unclear risk of bias [14,15,16,17,20,23].

With the exception of the study by Ball et al. [20], all included trials exhibited either an unclear or high risk of bias in several domains, including random sequence generation, allocation concealment, blinding of participants and personnel, and blinding of outcome assessment (Figure 2). Regarding incomplete outcome data, only the study by Ball et al. [20] exhibited a high risk of bias, whereas the remaining studies had a low risk. All studies had a low risk of bias for selective reporting.

### 3.4. Meta-Analysis Findings

**Figure 3:**
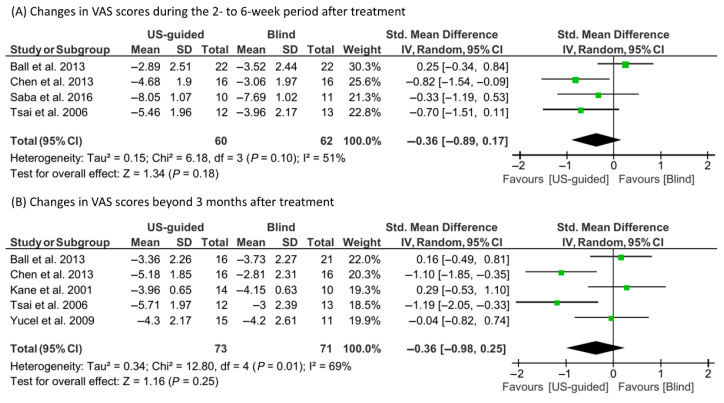
Results of the meta-analysis concerning changes in visual analog scale (VAS) scores: (**A**) at 2–6 weeks [14,15,16,20] and (**B**) at ≥12 weeks post-treatment [14,15,17,20,23].

A random-effect model was utilized due to observed heterogeneity (I^2^ = 51% and 69%, respectively) to evaluate the changes in VAS scores at 2–6 weeks and ≥12 weeks after corticosteroid injections (US-guided or blind) (Figure 3). The reduction in VAS scores in the US-guided group was not significantly different from that in the blind injection group at either 2–6 weeks (SMD = −0.36; 95% CI, −0.89 to 0.17; *p* = 0.18) or ≥12 weeks (SMD = −0.36; 95% CI, −0.98 to 0.25; *p* = 0.25).

**Figure 4:**
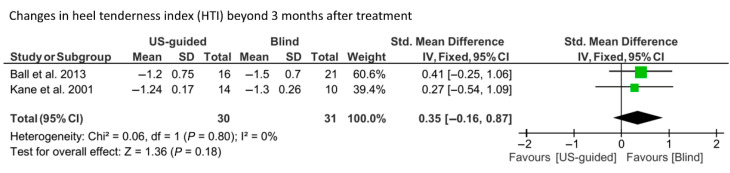
Results of the meta-analysis concerning changes in the heel tenderness index (HTI) scores at ≥12 weeks post-treatment [17,20].

A fixed-effect model was used due to the absence of heterogeneity (I^2^ = 0%) for the analysis of HTI scores at ≥12 weeks post-treatment (Figure 4). No significant difference was found in HTI score changes between the groups (SMD = 0.35; 95% CI, −0.16 to 0.87; *p* = 0.18).

**Figure 5:**
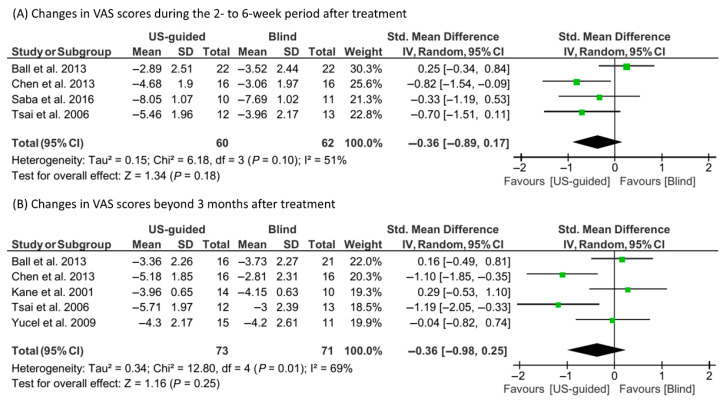
Results of the meta-analysis concerning changes in tenderness threshold (TT): (**A**) at 2–6 weeks [14,15] and (**B**) at ≥12 weeks post-treatment [14,15].

Changes in TT were assessed using a fixed-effect model at both time points (2–6 weeks: I^2^ = 0%; ≥12 weeks: I^2^ = 0%) (Figure 5). The US-guided injection group demonstrated a significantly greater increase in TT compared with the blind injection group at both 2–6 weeks (SMD = 1.01; 95% CI, 0.45 to 1.56; *p* < 0.01) and ≥12 weeks post-treatment (SMD = 1.53; 95% CI, 0.93 to 2.13; *p* < 0.01).

**Figure 6:**
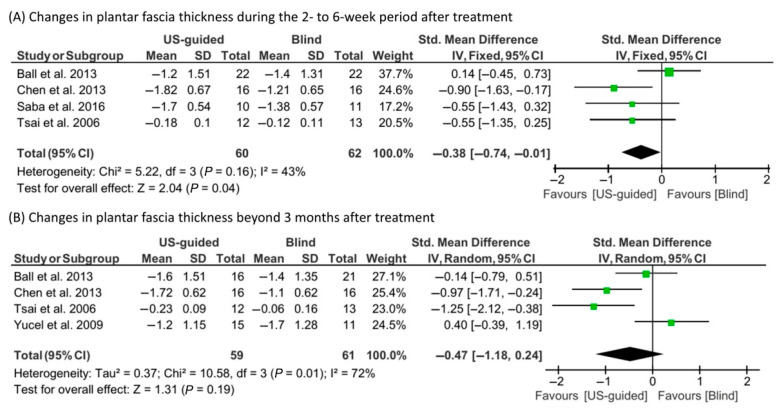
Results of the meta-analysis concerning changes in plantar fascia thickness: (**A**) at 2–6 weeks [14,15,16,20] and (**B**) at ≥12 weeks post-treatment [14,15,20,23].

In the meta-analysis of plantar fascia thickness, a fixed-effect model was utilized for the 2–6 week follow-up (I^2^ = 43%), while a random-effect model was used for the ≥12 week follow-up (I^2^ = 72%) (Figure 6). The US-guided injection group showed a significantly greater reduction in plantar fascia thickness at 2–6 weeks (SMD = −0.38; 95% CI, −0.74 to −0.01; *p* = 0.04). However, no significant difference was observed at 12 weeks (SMD = −0.47; 95% CI, −1.18 to 0.24; *p* = 0.19).

### 3.5. Publication Bias

**Figure 7:**
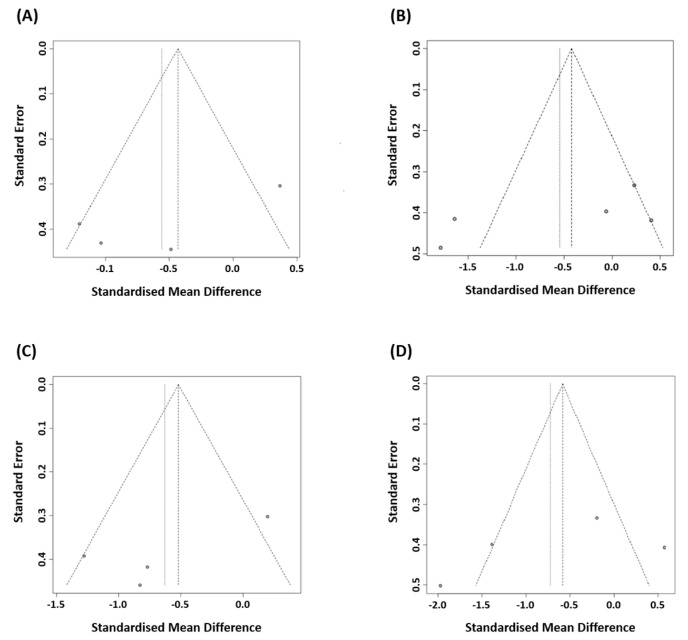
Funnel plots of the included studies: changes in visual analog scale (VAS) scores (**A**) at 2–6 weeks and (**B**) at ≥12 weeks post-treatment and changes in plantar fascia thickness (**C**) at 2–6 weeks and (**D**) at ≥12 weeks post-treatment.

Funnel plot analyses and Egger’s regression tests were conducted to assess potential publication bias for the changes in VAS scores and plantar fascia thickness at both 2–6 weeks and ≥12 weeks after treatment. The funnel plots appeared visually symmetrical (Figure 7). Egger’s test yielded *p*-values > 0.05 in all cases, indicating no significant publication bias. Specifically, the *p*-values for the changes in VAS scores were 0.21 at 2–6 weeks and 0.22 at ≥12 weeks, whereas the *p*-values for the changes in plantar fascia thickness were 0.18 and 0.43, respectively. Thus, there was no statistically significant evidence of publication bias.

## 4. Discussion

This meta-analysis evaluated the comparative efficacy of US-guided versus blind corticosteroid injections in PF treatment. Pain improvement was assessed using three common outcome measures: the VAS, HTI, and TT. No significant differences were observed between the groups in VAS and HTI scores. In contrast, TT—an indicator of mechanical pain tolerance—was significantly higher in the US-guided group at both short-term (2–6 weeks) and long-term (≥12 weeks) follow-ups. These differing results may reflect variations in the objectivity of the pain assessment tools. VAS is a fully subjective measure based on patients’ self-reported pain, which is susceptible to psychological and contextual influences, including emotional state [24], past experiences [25], cultural background [26,27], and cognitive appraisal [28]. Despite utilizing a semi-quantitative ordinal scale, HTI remains only partially standardized due to variability in the pressure applied by examiners and reliance on manual palpation, which introduces inter-examiner variability and patient-related bias. In contrast, TT is a quantitative measure obtained using pressure algometers that apply controlled mechanical force to determine the minimum pressure that induces pain [14,15,21]. This approach reduces examiner-related variability while enhancing reproducibility. Accordingly, TT would represent a more reliable and discriminative indicator of localized mechanical pain improvement, suggesting a potential clinical benefit of US-guided corticosteroid injections over the blind technique in patients with PF.

Plantar fascia thickness is a valid ultrasonographic measure used to objectively assess structural changes and treatment outcomes in PF [29,30,31]. In this analysis, US-guided corticosteroid injections were associated with a greater reduction in plantar fascia thickness compared to blind injections at the 2–6 week follow-up. This might be attributed to the superior anatomical precision of US guidance, which facilitates accurate localization of the pathological site and targeted corticosteroid delivery [12,14]. Accurate injection can reduce inflammation-related edema and fibrosis, contributing to the observed reduction in plantar fascia thickness [32,33]. Moreover, US guidance helps avoid off-target deposition of the medication.

Despite the inclusion of several RCTs, the overall methodological quality of the included studies was relatively low. Most studies exhibited either an unclear or high risk of bias in key domains, including random sequence generation, allocation concealment, and blinding of participants, personnel, and outcome assessors. Only one study (Ball et al. [20]) showed a low risk of bias in these areas. Therefore, the strength of the evidence is limited, requiring caution while interpreting the findings.

Moreover, significant heterogeneity was observed across studies in terms of corticosteroid type and dosage, which likely influenced local pharmacodynamics, anti-inflammatory potency, and duration of effect and contributed to variability in clinical outcomes. Furthermore, injection targets and approach methods were different across the included studies. In the US-guided group, Ball et al. [20] and Tsai et al. [14] used a posterior approach aligned with the long axis of the transducer, targeting the superficial border of the plantar fascia enthesis and the thickened proximal fascia, respectively. Kane et al. [17], Saba et al. [16], and Yucel et al. [23] employed a medial approach, directing the injection toward areas of maximal sonographic abnormality, while Chen et al. [15] targeted the origin of the plantar fascia. In the blind group, Ball et al. [20] used a posterior approach, inserting the needle parallel to the heel pad toward the medial calcaneal tubercle. In contrast, Kane et al. [17], Saba et al. [16], and Yucel et al. [23] adopted a medial approach, targeting the point of maximal tenderness identified by palpation. Furthermore, Tsai et al. [14] used an anteromedial approach toward the most tender region near the calcaneal tuberosity. These inconsistencies in injection targets and approach methods might have impacted injection accuracy, corticosteroid distribution, and ultimately, clinical effectiveness. Our meta-analysis did not consider the various aforementioned factors that could influence treatment outcomes, primarily due to the limited number of included studies.

The findings of this meta-analysis underscore the potential benefits of US-guided corticosteroid injections as an alternative to blind injections in patients with PF. However, given the generally low methodological quality and heterogeneity of existing studies, the clinical implementation of routine US-guided injection should be considered with caution. Future high-quality RCTs that standardize corticosteroid type, dosage, injection targets, and techniques for both US-guided and blind injections in patients with PF are needed to establish a solid basis for clarifying the relative effectiveness of US-guided versus blind corticosteroid injection approaches.

## 5. Conclusions

Based on the results of this meta-analysis, US-guided injections provided superior pain relief, as evidenced by improvements in TT in both the short- and long-term periods, along with a greater reduction in plantar fascia thickness at short-term follow-up. However, no significant differences were observed between the two groups in pain outcomes as measured by the VAS and HTI. Given the relatively low methodological quality of the included studies, these findings should be interpreted with caution. Thus, further high-quality RCTs are needed to clarify the potential therapeutic advantages of US-guided corticosteroid injection.

## Figures and Tables

**Table 1:** Characteristics of the included studies.

Study	Study Design	Number of Treated Feet (US-Guided/Blind)	Post-Treatment Follow-Up	Outcome Measures
Ball et al. [20] 2013	RCT	22/22	6 and 12 weeks	VAS, HTI, plantar fascia thickness
Chen et al. [15] 2013	RCT	16/16	3 weeks and 3 months	VAS, TT, plantar fascia thickness, heel pad thickness, incidence of hypoechogenic fascia
Kane et al. [17] 2001	RCT	14/10	Mean 13.4 weeks (range 6–48 weeks)	VAS, HTI
Saba et al. [16] 2016	RCT	10/11	2 and 4 weeks	VAS, plantar fasciitis pain/disability scale, plantar fascia thickness, plantar fascia echogenicity, clinical remission, ultrasonographic remission, clinical and ultrasonographic remission
Tsai et al. [14] 2006	RCT	12/13	2 weeks, 2 months, and 1 year	VAS, TT, plantar fascia thickness, hypoechogenicity
Yucel et al. [23] 2009	RCT	15/11	Mean 25.3 months (range 22.2–27.2 months)	VAS, plantar fascia thickness, fat pad thickness, fascial hypoechogenicity

Abbreviations: RCT, randomized controlled trial; VAS, visual analog scale; HTI, heel tenderness index; TT, tenderness threshold.

## Data Availability

The datasets used and analyzed during the current study are available from the corresponding author on reasonable request.

## Supplementary Materials

The following supporting information can be downloaded at: https://www.mdpi.com/article/10.3390/life15071107/s1.

## Abbreviations

US	Ultrasound
PF	Plantar fasciitis
VAS	Visual analog scale
HTI	Heel tenderness index
TT	Tenderness threshold
SMDs	Standardized mean differences
CIs	Confidence intervals
RCTs	Randomized controlled trials
