# Supplementary material for: Comparison of Effectiveness Between Ultrasound-Guided and Blind Corticosteroid Injections in Plantar Fasciitis: A Systematic Review and Meta-Analysis
Source: Life (Basel). 2025 Jul 15;15(7):1107. doi: 10.3390/life15071107 (PMC12298417; doi:10.3390/life15071107)
Supplement: Supplementary file 1 [file life-15-01107-s001.zip › life-3714234-supplementary.pdf]

Search Strategy

A comprehensive literature search was conducted across four electronic databases: PubMed, Embase, Web of Science and Scopus.

PubMed:

The search strategy used was as follows:

(("Plantar Fasciitis"[All Fields] OR "Plantar Fascia"[All Fields] OR "Plantar Fasciopathy"[All Fields] OR "Heel"[All Fields] OR "Heel Pain"[All Fields]) AND ("Ultrasound"[All Fields] OR "Ultrasonography"[All Fields] OR "Sonography"[All Fields] OR "Ultrasound-Guided"[All Fields]) AND ("Ultrasound-Guided Injection"[All Fields] OR "Injection"[All Fields] OR "Corticosteroid Injection"[All Fields] OR "Plantar Fascia Injection"[All Fields] OR "Corticosteroid"[All Fields] OR "Saline Solution"[All Fields] OR "Placebo"[All Fields] OR "Local Anesthetics"[All Fields] OR "OrthoBiologics"[All Fields] OR "Platelet-Rich Plasma"[All Fields] OR "Whole Blood"[All Fields] OR "Mesenchymal Stem Cells"[All Fields] OR "Amnion"[All Fields] OR "Adipose Tissue"[All Fields] OR "Fat Injection"[All Fields] OR "Dextrose"[All Fields] OR "Botulinum Toxins"[All Fields] OR "Fasciotomy"[All Fields] OR "Tenotomy"[All Fields] OR "Tenex"[All Fields] OR "Prolotherapy"[All Fields] OR "Electrolysis"[All Fields] OR "High-Energy Shock Waves"[All Fields] OR "Palpation"[All Fields] OR "Anatomic Landmarks"[All Fields] OR "Physical Therapy"[All Fields])) AND ((humans[Filter]) AND (english[Filter]))

Embase:

The Embase search included both exploded subject headings and free-text terms:

#1 : ('plantar fasciitis'/exp OR 'plantar fasciitis' OR 'plantar fascia'/exp OR 'plantar fascia' OR 'plantar fasciopathy'/exp OR 'plantar fasciopathy' OR 'heel'/exp OR 'heel' OR 'heel pain'/exp OR 'heel pain') AND ('ultrasound'/exp OR 'ultrasound' OR 'ultrasonography'/exp OR 'ultrasonography' OR 'sonography'/exp OR 'sonography' OR 'ultrasound-guided') AND ('ultrasound-guided injection' OR 'injection'/exp OR 'injection' OR 'corticosteroid injection' OR 'plantar fascia injection' OR 'corticosteroid'/exp OR 'corticosteroid' OR 'saline solution'/exp OR 'saline solution' OR 'placebo'/exp OR 'placebo' OR 'local anesthetics' OR 'orthobiologics'/exp OR 'orthobiologics' OR 'platelet-rich plasma'/exp OR 'platelet-rich plasma' OR 'whole blood'/exp OR 'whole blood' OR 'mesenchymal stem cells'/exp OR 'mesenchymal stem cells' OR 'amnion'/exp OR 'amnion' OR 'adipose tissue'/exp OR 'adipose tissue' OR 'fat injection'/exp OR 'fat injection' OR 'dextrose'/exp OR 'dextrose' OR 'botulinum toxins'/exp OR 'botulinum toxins' OR 'fasciotomy'/exp OR 'fasciotomy' OR 'tenotomy'/exp OR 'tenotomy' OR 'tenex'/exp OR 'tenex' OR 'prolotherapy'/exp OR 'prolotherapy' OR 'electrolysis'/exp OR 'electrolysis' OR 'high-energy shock waves'/exp OR 'high-energy shock waves' OR 'palpation'/exp OR 'palpation' OR 'anatomic landmarks'/exp OR 'anatomic landmarks' OR 'physical therapy'/exp

OR 'physical therapy') AND [humans]/lim AND [english]/lim

#2 : #1 AND [embase]/lim NOT ([embase]/lim AND [medline]/lim)

Web of Science:

The search was conducted using the following terms in All Text:

(("Plantar Fasciitis" OR "Plantar Fascia" OR "Plantar Fasciopathy" OR "Heel" OR "Heel Pain") AND ("Ultrasound" OR "Ultrasonography" OR "Sonography" OR "Ultrasound-Guided")) AND ("Ultrasound-Guided Injection" OR "Injection" OR "Corticosteroid Injection" OR "Plantar Fascia Injection" OR "Corticosteroid" OR "Saline Solution" OR "Placebo" OR "Local Anesthetics" OR "OrthoBiologics" OR "Platelet-Rich Plasma" OR "Whole Blood" OR "Mesenchymal Stem Cells" OR "Amnion" OR "Adipose Tissue" OR "Fat Injection" OR "Dextrose" OR "Botulinum Toxins" OR "Fasciotomy" OR "Tenotomy" OR "tenax" OR "Prolotherapy" OR "Electrolysis" OR "High-Energy Shock Waves" OR "Palpation" OR "Anatomic Landmarks" OR "Physical Therapy") (All Fields)

Scopus:

The search was conducted using the TITLE-ABS-KEYfield, which includes the Title, Abstract, and Keywords.

TITLE-ABS-KEY ((("Plantar Fasciitis" OR "Plantar Fascia" OR "Plantar Fasciopathy" OR "Heel" OR "Heel Pain") AND ("Ultrasound" OR "Ultrasonography" OR "Sonography" OR "Ultrasound-Guided")) AND ("Ultrasound-Guided Injection" OR "Injection" OR "Corticosteroid Injection" OR "Plantar Fascia Injection" OR "Corticosteroid" OR "Saline Solution" OR "Placebo" OR "Local Anesthetics" OR "OrthoBiologics" OR "Platelet-Rich Plasma" OR "Whole Blood" OR "Mesenchymal Stem Cells" OR "Amnion" OR "Adipose Tissue" OR "Fat Injection" OR "Dextrose" OR "Botulinum Toxins" OR "Fasciotomy" OR "Tenotomy" OR "Tenex" OR "Prolotherapy" OR "Electrolysis" OR "High-Energy Shock Waves" OR "Palpation" OR "Anatomic Landmarks" OR "Physical Therapy"))
